# Gene Coexpression Connectivity Predicts Gene Targets Underlying High Ionic-Liquid Tolerance in Yarrowia lipolytica

**DOI:** 10.1128/msystems.00348-22

**Published:** 2022-07-12

**Authors:** Caleb Walker, Seunghyun Ryu, Sergio Garcia, David Dooley, Brian Mendoza, Cong T. Trinh

**Affiliations:** a Department of Chemical and Biomolecular Engineering, University of Tennessee, Knoxville, Tennessee, USA; University of Waterloo

**Keywords:** *Yarrowia lipolytica*, oleaginous yeast, ionic liquid, solvent tolerance, robustness, dynamic RNA-Seq, gene coexpression connectivity, GeCCo, gene targets

## Abstract

Microbial tolerance to organic solvents such as ionic liquids (ILs) is a robust phenotype beneficial for novel biotransformation. While most microbes become inhibited in 1% to 5% (vol/vol) IL (e.g., 1-ethyl-3-methylimidazolium acetate), we engineered a robust Yarrowia lipolytica strain (YlCW001) that tolerates a record high of 18% (vol/vol) IL via adaptive laboratory evolution. Yet, genotypes conferring high IL tolerance in YlCW001 remain to be discovered. In this study, we shed light on the underlying cellular processes that enable robust Y. lipolytica to thrive in inhibitory ILs. By using dynamic transcriptome sequencing (RNA-Seq) data, we introduced Gene Coexpression Connectivity (GeCCo) as a metric to discover genotypes conferring desirable phenotypes that might not be found by the conventional differential expression (DE) approaches. GeCCo selects genes based on their number of coexpressed genes in a subnetwork of upregulated genes by the target phenotype. We experimentally validated GeCCo by reverse engineering a high-IL-tolerance phenotype in wild-type Y. lipolytica. We found that gene targets selected by both DE and GeCCo exhibited the best statistical chance at increasing IL tolerance when individually overexpressed. Remarkably, the best combination of dual-overexpression genes was genes selected by GeCCo alone. This nonintuitive combination of genes, BRN1 and OYE2, is involved in guiding/regulating mitotic cell division, chromatin segregation/condensation, microtubule and cytoskeletal organization, and Golgi vesicle transport.

**IMPORTANCE** Cellular robustness to cope with stressors is an important phenotype. Y. lipolytica is an industrial robust oleaginous yeast that has recently been discovered to tolerate record high concentrations of ILs, beneficial for novel biotransformation in organic solvents. However, genotypes that link to IL tolerance in Y. lipolytica are largely unknown. Due to the complex IL-tolerant phenotype, conventional gene discovery and validation based on differential gene expression approaches are time-consuming due to a large search space and might encounter a high false-discovery rate. Here, using the developed Gene Coexpression Connectivity (GeCCo) method, we identified and validated a subset of most promising gene targets conferring the IL-tolerant phenotypes and shed light on their potential mechanisms. We anticipate GeCCo being a useful method to discover the genotype-to-phenotype link.

## INTRODUCTION

Cellular robustness such as solvent tolerance is an important phenotype in natural and engineered biological systems. Biocatalysis in organic solvents enables novel biotransformation with unique strategies for substrate solubilization (1), enhanced enzymatic activity (2), and product recovery (3). Ionic liquids (ILs) have recently emerged as green organic solvents to replace the conventional ones in bioprocessing (4). These solvents can provide a novel reaction medium for biotransformation with superior performance due to their ability to dissolve a broader range of compounds and their adjustable properties for enzyme stabilization and activation (e.g., lipases, alcohol dehydrogenases, proteases, and oxidoreductases) (5). Some ILs such as 1-ethyl-3-methylimidazolium acetate ([EMIM][OAc]) are promising for the cellulosic biomass pretreatment technology that effectively reduces recalcitrance, achieving efficient dissolution of biomass and permitting enzymatic accessibility to the sugar polymers (i.e., cellulose and hemicellulose) for saccharification (6–8). Fermentative sugars released from IL-pretreated biomass could be assimilated by microbial cell factories if not for the toxicity that ILs impose on microorganisms (9, 10). For these reasons, microbial high solvent tolerance is a desirable phenotype for conversion of renewable cellulosic biomass to replace petroleum-derived chemicals and fuels.

ILs are toxic to most industrial workhorse microorganisms (e.g., Escherichia coli and Saccharomyces cerevisiae). Cell growth is inhibited even at low IL concentrations, for example, 1 to 5% (vol/vol) [EMIM][OAc] (11, 12). These ILs disrupt cell membranes and intracellular processes (13–15). Efflux pumps have been reported to improve IL tolerance in microorganisms (9, 16–19). Currently, knowledge of novel genotypes responsible for IL tolerance in microorganisms is still very limited. Remarkably, screens for IL-tolerant microorganisms identified the GRAS (generally regarded as safe) oleaginous yeast Yarrowia lipolytica as one of the top performers in the benchmark [EMIM][OAc] (20). Wild-type (WT) Y. lipolytica could achieve 92% of the theoretical yield of alpha-ketoglutaric acid (KGA) in 10% (vol/vol) [EMIM][OAc] during simultaneous saccharification and fermentation of IL-pretreated cellulose (21). Due to its robustness and broad industrial use, Y. lipolytica is a promising model organism to study IL tolerance.

To understand the robustness of Y. lipolytica in ILs, adaptive laboratory evolution (ALE) was performed to generate a platform strain, YlCW001, with enhanced activity in high concentrations of 18% (vol/vol) [EMIM][OAc] (22). Remarkably, the mutant (MT) also outperformed the wild-type Y. lipolytica in all imidazolium-based ILs tested. Physiological, metabolic, and genetic characterization elucidated that sterols are critical for IL tolerance in Y. lipolytica by strengthening the cell membrane (22). Despite the discovery of the beneficial role of sterols, other novel genotypes that confer IL tolerance in Y. lipolytica still remain to be discovered. Even though genome-wide analysis of the wild-type and evolved mutant Y. lipolytica could provide evidence of genotypes that may be involved in the phenotypic variance in IL tolerance, it is very challenging to identify and validate gene targets and their combinations among a large set of gene candidates in response to high IL exposure. Since IL tolerance is a complex phenotype, the most differentially expressed genes identified by omics data (e.g., transcriptome sequencing [RNA-Seq]) alone might not necessarily be the prime candidates conferring IL tolerance.

In this study, we seek to identify the key genotype(s) conferring IL tolerance in Y. lipolytica. Using dynamic transcriptomic data of the wild-type and evolved Y. lipolytica strains growing with and without IL, we shed light on the underlying cellular processes that Y. lipolytica uses to respond to IL. We further developed Gene Coexpression Connectivity (GeCCo) as a metric to identify a small subset of promising gene targets conferring IL tolerance among a large set of potential gene candidates. Specifically, GeCCo selects the most connected genes from a coexpression network of upregulated genes. By performing single- and dual-gene overexpression validation, we demonstrated that GeCCo could reveal the key genotype(s) behind the complex phenotype(s) that was reverse engineered to achieve high solvent tolerance in Y. lipolytica.

## RESULTS

### Modified genotype of the IL-tolerant evolved strain YlCW001.

**FIG 1:**
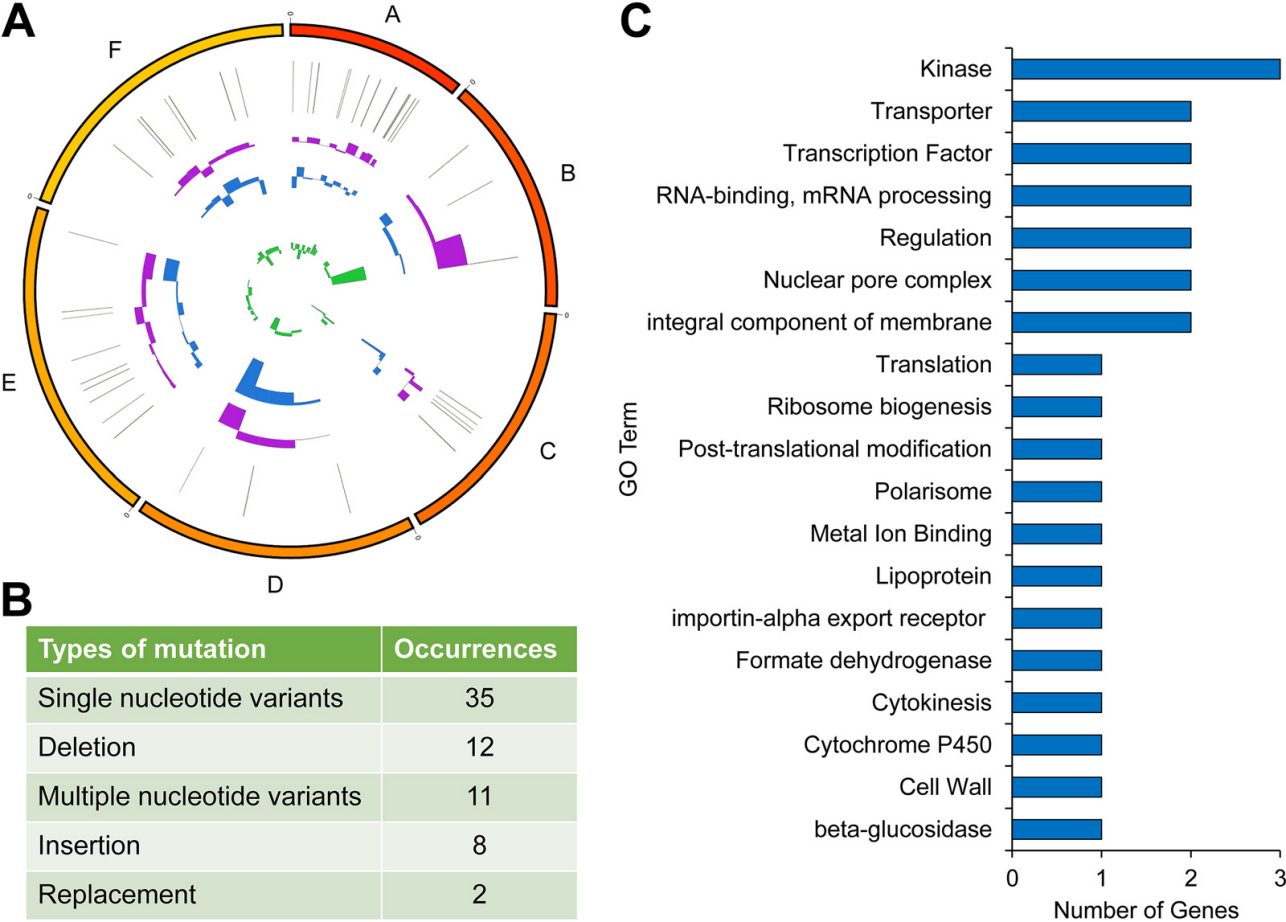
Mutated genes of IL-tolerant mutant Y. lipolytica (MT, YlCW001), which include 68 variants across 40 genes that change amino acid residues. (A) Chromosomal location of mutated genes depicting log_2_ fold changes at the mid-exponential RNA-seq sample between the MT in 8% [EMIM][OAc] and WT in 0% IL (purple), WT in 8% IL (blue), and MT in 0% IL (green). (B) Types of variants in the mutated genes. (C) Gene ontology of the mutated genes.

To connect genetic mutations with enhanced IL tolerance, we resequenced the evolved strain YlCW001 (MT) and analyzed the mutations. Across the entire genome, we detected a total of 648 variants, but only 40 genes contained variants that caused an amino acid change (Fig. 1A; see also Data Set S1 in the supplemental material). These 40 mutated genes (MGs) are randomly distributed across the 6 chromosomes of Y. lipolytica and hold a total of 68 mutations (i.e., variants) including 35 single nucleotide variants (SNV), 12 deletions, 11 multiple nucleotide variants (MNV), 8 insertions, and 2 replacements (Fig. 1B). The most MGs were in chromosome A (13 MGs) followed by chromosome E (9 MGs), chromosome F (7 MGs), chromosome C (6 MGs), and chromosomes B and D (each with 3 MGs) (Fig. 1A). By performing gene ontology (GO) associations for the 40 MGs using ClueGo, Integrated Microbial Genomes (IMG), and BLASTp against S. cerevisiae, we were able to assign the functional annotations for 68% of the MGs (Fig. 1C). Kinases represent the most abundant GO terms, containing 3 MGs, followed by transporters, transcription factors, mRNA processing, regulation, nuclear pore complex, and membrane components (2 MGs each; see Data Set S1 for gene identifiers [IDs]).

10.1128/msystems.00348-22.7 DATA SET S1 A list of genetic mutations of the evolved strain YlCW001. Download Data Set S1, XLSX file, 0.1 MB. Copyright © 2022 Walker et al. 2022 Walker et al. https://creativecommons.org/licenses/by/4.0/ This content is distributed under the terms of the Creative Commons Attribution 4.0 International license.

### Basal gene expression differences between the WT and MT strains without IL exposure.

**FIG 2:**
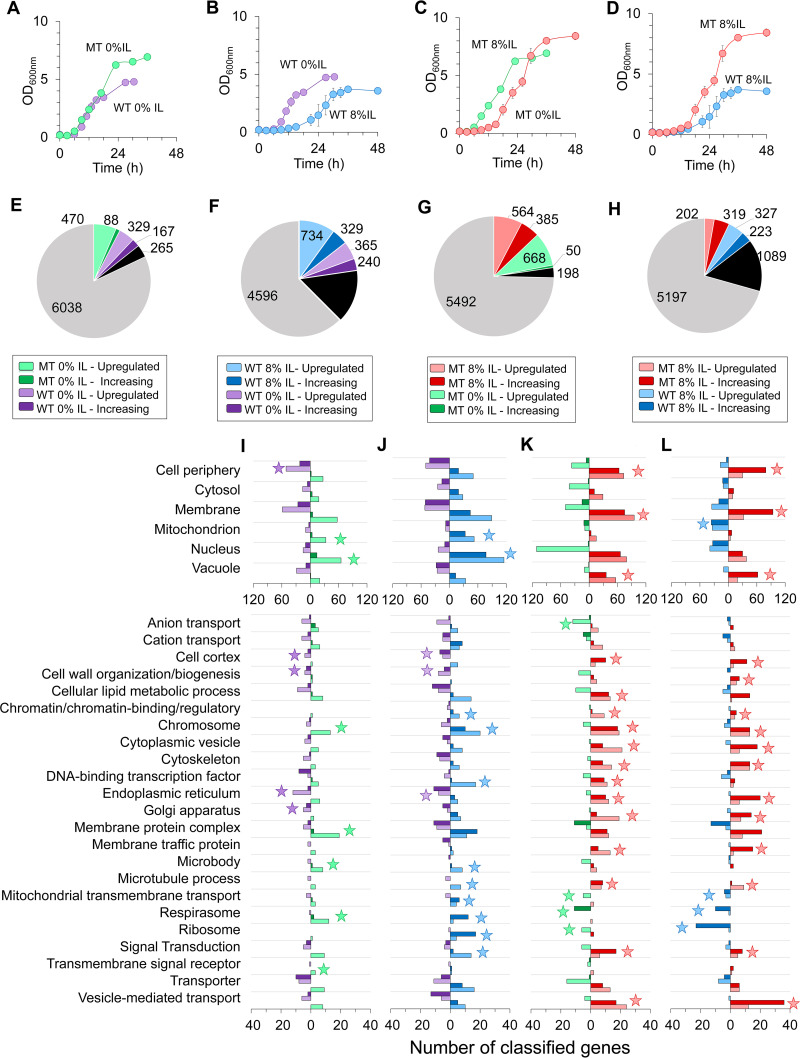
(A to D) Growth profiles of MT and WT in 0% IL (A), WT in 0% IL and WT in 8% (vol/vol) IL (B), MT in 0% IL and MT in 8% (vol/vol) IL (C), and growth of WT and MT in 8% (vol/vol) IL (D). (E to H) Number and distribution of classified genes for MT in 0% IL versus WT in 0% IL (E), WT in 8% IL versus WT in 0% IL (F), MT in 8% IL versus MT in 0% IL (G), and MT in 8% IL versus WT in 8% IL (H). In these pie charts, black and gray pies refer to “changed regulation” and “no change” genes, respectively. (I to L) Classified gene ontology associations for basal differences in gene expression between the MT and WT in 0% IL (I), IL-responsive gene expression of the WT strain (J), IL-responsive gene expression of the MT strain (K), and enhanced IL-responsive gene expression of the MT strain versus the WT strain in 8% IL (L). Stars represent ontology terms where the sum of upregulated and increasing genes under a biological condition is at least twice that under the other biological condition.

We first examined the basal changes in gene expression between the WT and MT strains growing in medium without IL (Fig. 2A). Temporal RNA-Seq data for both the WT and MT strains were collected at the early and mid-exponential growth phases and subjected to the gene classification analysis (Data Set S2, tab 1). We identified 470 “upregulated” genes and 88 “increasing” genes in the MT strain relative to the WT strain cultured without IL (Fig. 2E). Here, upregulated genes were overexpressed at both early and mid-exponential phases while increasing genes were overexpressed at mid-exponential phase but do not exhibit significant change at early exponential phase (see Materials and Methods). These genes with greater expression in the MT strain were annotated for mitochondrion, nucleus, chromosome, membrane protein complex, microbody, respirasome, and transmembrane signal receptors (Fig. 2I). For the WT strain, we found 329 upregulated genes and 167 increasing genes relative to the MT strain cultured without IL. These genes were involved in cell periphery, cell cortex, cell wall organization/biogenesis, endoplasmic reticulum, and the Golgi apparatus (Fig. 2I). Five of the MGs were differentially expressed between the MT and WT cultured without IL. The MT upregulated 3 of these MGs including an RNA metabolic protein (REH1, YALI0B08734g), an RNA splicing factor (WHI1, YALI0F12375g), and an uncharacterized protein (YALI0C13002g). Meanwhile, the MT downregulated 2 of these MGs, an expansin-like protein (YALI0E17941g) and a nonreceptor serine/threonine protein kinase (SKY1, YALI0A18590g).

10.1128/msystems.00348-22.8 DATA SET S2 Gene classification for (i) mutant in 0% IL versus wild type in 0% IL, (ii) wild type in 0% IL versus wild type in 8% IL, (iii) mutant in 0% IL versus mutant in 8% IL, and (iv) mutant in 8% IL versus wild type in 8% IL. Download Data Set S2, XLSX file, 1.4 MB. Copyright © 2022 Walker et al. 2022 Walker et al. https://creativecommons.org/licenses/by/4.0/ This content is distributed under the terms of the Creative Commons Attribution 4.0 International license.

### IL-responsive gene expression. (i) Wild-type response to IL.

Next, we aimed to understand gene expression changes for the WT strain exposed to IL (Fig. 2B) (Data Set S2, tab 2). This RNA-seq pairwise set exhibited the greatest perturbation of gene expression relative to all other data sets with only ~62% of genes classified as no change (Fig. 2F). We found 734 upregulated genes and 329 increasing genes for the WT growing in 8% (vol/vol) IL. These IL-induced genes were enriched for mitochondrion (including mitochondrial transmembrane transport and respirasome), genetic processing (i.e., nucleus, chromosome, chromatin binding/regulation, DNA-binding transcription factors, ribosome, and signal transduction), microtubule processing, and microbody (Fig. 2J). In contrast, we identified 365 upregulated genes and 240 increasing genes for the WT cultured without IL. However, only 3 annotated processes were enriched more than 2-fold, which included cell cortex, cell wall organization/biogenesis, and the endoplasmic reticulum (Fig. 2J).

### (ii) Mutant response to IL.

Likewise, we compared gene expression changes for the MT strain exposed to IL (Fig. 2C) (Data Set S2, tab 3). The gene classification analysis identified 564 upregulated genes and 385 increasing genes for the MT strain growing in 8% (vol/vol) IL relative to the MT strain without IL (Fig. 2G). These IL-induced genes for the MT strain were enriched for lipid processes (i.e., membrane, cellular lipid metabolic process, membrane traffic protein, Golgi apparatus, and endoplasmic reticulum), cell periphery (including cell cortex, cytoskeleton, and microtubule process), intracellular vesicles (i.e., vacuole, cytoplasmic vesicle, and vesicle-mediated transport), and genetic processes (i.e., chromosome, chromatin/chromatin binding/regulator, DNA-binding transcription factor, and signal transduction) (Fig. 2K). The MT strain cultured without IL exhibited 668 upregulated genes and only 50 increasing genes. These gene sets were annotated for anion and mitochondrial transmembrane transport, respirasome, and the ribosome (Fig. 2K). We also identified 9 of the MGs among the classified genes. Of these, 5 were upregulated in IL including an actin cytoskeletal protein (YALI0A18381g), a nonreceptor serine/threonine protein kinase (CBK1, YALI0B04268g), a cell septum/cytosolic protein (ZDS2, YALI0F12793g), an exportin transporter (CSE1, YALI0E07139g), and an uncharacterized protein (YALI0B18194g). One MG was downregulated in IL, a protein component of the polarisome (SPA2, YALI0F16665g), and one uncharacterized protein (YALI0F08107g) was classified with changed regulation in IL.

### (iii) Enhanced IL response by the mutant.

Our gene classification of time series RNA-seq data revealed 202 upregulated genes and 319 increasing genes for the MT strain in IL relative to the WT strain in IL (Fig. 2D and H) (Data Set S2, tab 4). These genes were associated with lipid processes (i.e., membrane, membrane traffic protein, Golgi apparatus, and endoplasmic reticulum), cell periphery (including cell cortex, cytoskeleton, cell wall organization/biogenesis, and microtubule process), intracellular vesicles (i.e., vacuole, cytoplasmic vesicle, and vesicle-mediated transport) and genetic processes (i.e., chromosome, chromatin/chromatin binding/regulator, and signal transduction) (Fig. 2L). In contrast, the WT exhibited 327 upregulated genes and 223 increasing genes relative to the MT when both strains were cultured in IL. These genes were associated with mitochondrion, mitochondrial transmembrane transport, respirasome, and the ribosome (Fig. 2L). We found that 8 of the MGs were identified in the classified genes. The MT upregulated an exportin transporter (CSE1, YALI0E07139g), and 3 genes were classified as increasing, including a chaperone (YALI0A09108g), a translation initiation factor (RPG1, YALI0C16247g), and a protein component of the polarisome (SPA2, YALI0F16665g). Four of these MGs were classified with changed regulation, including an actin cytoskeletal protein (YALI0A18381g), a nonreceptor serine/threonine protein kinase (CDC7, YALI0F23287g), and uncharacterized proteins (YALI0A13233g and YALI0C13002g).

### Identifying genotypes conferring IL tolerance. (i) Choice of pairwise data set to select gene targets conferring IL tolerance.

In response to IL, there were 734 genes upregulated by the WT (Fig. 2F) and 564 genes upregulated by the MT (Fig. 2G). However, many of these differentially expressed genes are potentially a consequence of IL toxicity (i.e., indirect effect) as opposed to conferring IL tolerance (i.e., direct effect). Therefore, using the appropriate pairwise data set to select gene targets conferring IL tolerance is critical due to a large space of candidates and their combinations. Fortunately, the MT selected from ALE outperformed the WT when both were cultured in IL. It also possesses superior IL-tolerant genotypes. Taken altogether, we reasoned that, to identify genotypes conferring IL tolerance, the best gene candidates for reverse engineering high solvent tolerance could be found by comparing WT and MT gene expression in IL (Data Set S2, tab 4).

### (ii) Formulation of gene coexpression connectivity (GeCCo) as a metric to select gene targets for reverse engineering.

Conventionally, gene targets are selected based on greatest fold change between strains or conditions and/or gene ontology if the system is well understood. Using the pairwise data set of WT and MT in IL, our analysis identified 202 candidate gene targets that were upregulated across 2 time points by the MT (Fig. 2G). It is laborious to experimentally validate these genes for IL tolerance either in isolation or in combinations. To reduce the most probable candidate list of gene targets for validation, we introduced GeCCo, which selects highly connected and classified genes in the coexpression network.

**FIG 3:**
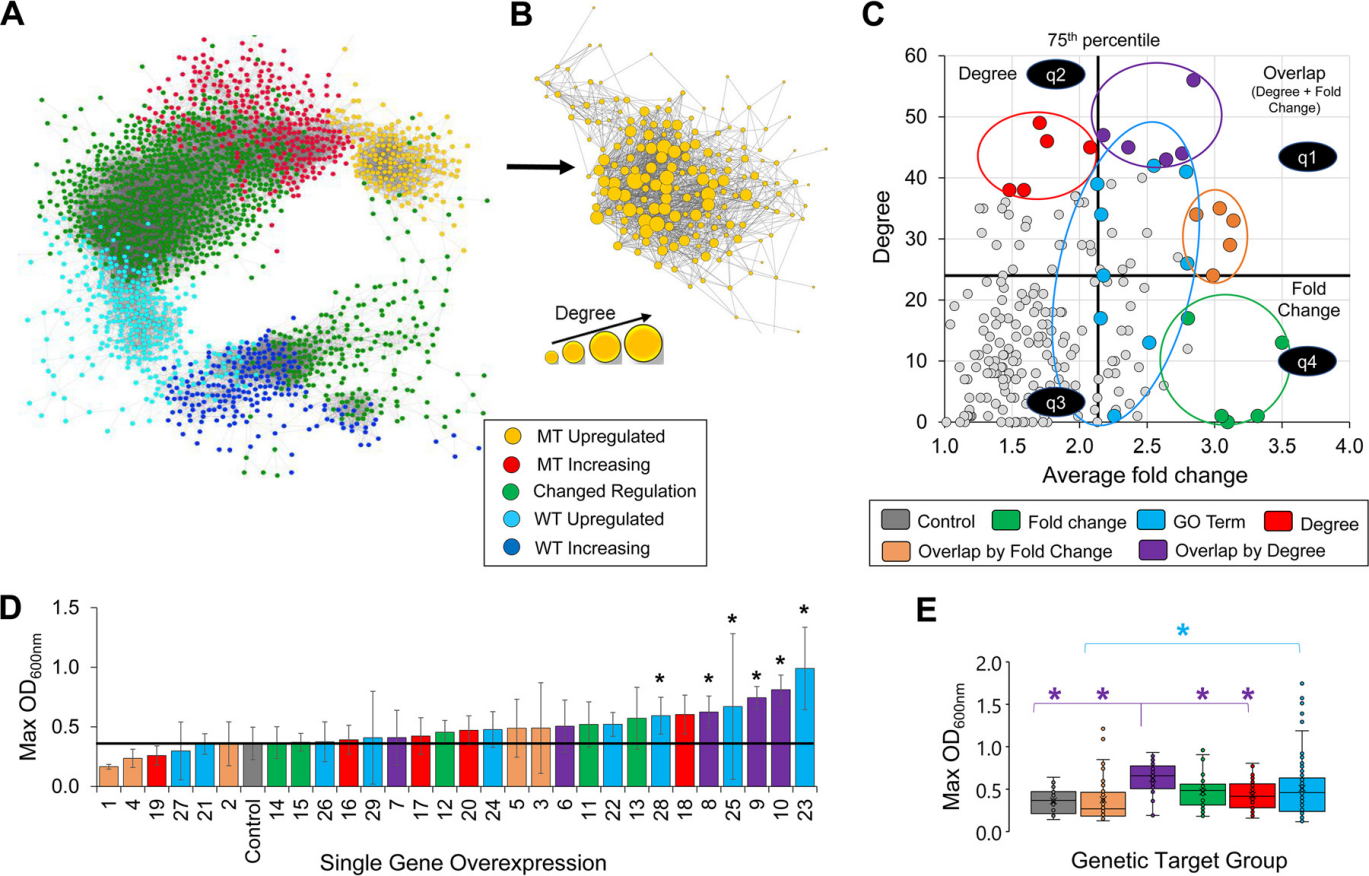
(A) Coexpression network of classified genes between the MT and WT in 8% IL. (B) Subnetwork of coexpressed genes upregulated by the MT in 8% IL, illustrating gene connectivity by degree. (C) Gene targets selected by degree, fold change, overlap by fold change, overlap by degree, or gene ontology. (D) Maximum cell growth for individual overexpression of gene targets in 11% IL. (E) Box-and-whisker plot for each group of gene targets depicting all replicate maximum OD_600_ values for all associated genes contained within each group. *. *P* value ≤ 0.05 (*n* ≥ 9 for each gene and *n* = 27 for the empty-vector control).

In the first step of GeCCo, the coexpression network using the Pearson correlation (23) is built for the genes differentially expressed and regulated between the MT and WT strains in IL. Each gene in the coexpression network is classified using the gene classification methodology (see Materials and Methods and Fig. S1). Figure 3A shows the network topology where each group of classified genes exhibited tight coexpression. For instance, genes that were upregulated or increased by the MT in IL were separated from genes that were upregulated or increased by the WT in IL. These gene clusters were connected by genes classified as changed regulation. In the second step, GeCCo selects the most connected genes in a subnetwork (e.g., upregulated gene subnetwork) based on their degree centrality. Here, degree centrality is the total number of edges connected to a vertex in the coexpression network. For instance, Fig. 3B showed the most connected genes in the subnetwork of genes upregulated by the MT in IL.

10.1128/msystems.00348-22.1 FIG S1 Gene classification methodology. (Step 1) For each pairwise set of biological conditions, gene TPM values are floored to a minimum of 5, averaged, and converted into log_2_ scale. (Step 2) Fold change (*X* and *Y*) and regulation (*Z*) scores are calculated between biological conditions. (Step 3) Genes are classified based on the scores of *X*, *Y*, and *Z* into upregulated or increasing for a biological condition, changed regulation, or no change. Download FIG S1, TIF file, 0.2 MB. Copyright © 2022 Walker et al. 2022 Walker et al. https://creativecommons.org/licenses/by/4.0/ This content is distributed under the terms of the Creative Commons Attribution 4.0 International license.

### (iii) Selection of gene targets by GeCCo for experimental validation.

To demonstrate GeCCo, we used the subnetwork of genes upregulated by the MT in IL. For each of the 202 genes upregulated by the MT, the average fold change between the 2 transcriptomic time points (i.e., *X* [early] and *Y* [mid] scores; see Fig. S1) and the degree of each gene from the coexpression subnetwork were calculated (Fig. 3B). These genes were divided into 4 quadrants based on the 75th percentile of average fold change and on the 75th percentile of degree (Fig. 3C). Quadrant 1 represents genes that were in the 75th percentile of both average fold change and degree, quadrant 2 represents genes that belong only in the 75th percentile of degree, quadrant 3 represents genes that were not in the 75th percentile of either degree or average fold change, and quadrant 4 represents genes that belong in the 75th percentiles of both degree and average fold change (Fig. 3C). To test GeCCo, we selected the top 5 genes based on degree (quadrant 2), the top 5 genes based on degree overlapping average fold change (i.e., overlap by degree, quadrant 1), and the top 5 genes based on average fold change overlapping degree (i.e., overlap by fold change, quadrant 1). For control, we chose the top 5 genes based on average fold change (quadrant 4) and 9 genes based on gene ontology associations related to membrane, transport, kinase, cell wall, actin cytoskeleton organization, and myosin complex (quadrants 1, 2, and 4) (Fig. 3C and Table S1).

10.1128/msystems.00348-22.2 TABLE S1 Gene target locus tag, accession number, average fold change, degree, category of selection, and ortholog of Saccharomyces cerevisiae. Download Table S1, XLSX file, 0.01 MB. Copyright © 2022 Walker et al. 2022 Walker et al. https://creativecommons.org/licenses/by/4.0/ This content is distributed under the terms of the Creative Commons Attribution 4.0 International license.

### Validation of gene targets conferring IL tolerance. (i) Single-gene overexpression.

Each of the 29 selected gene targets (Data Set S3) were tested for their efficacy in conferring IL tolerance by individually overexpressing each gene in the WT strain and characterizing cell growth in 11% IL (Fig. 3D). In this extremely harsh concentration of IL, genes chosen from overlap by degree exhibited the best cell growth in IL among all the other target groups except genes chosen by GO term (Fig. 3E). Of all the target groups, only 6 of the genes caused a decrease in cell mass, including 3 genes chosen by overlap by fold change (#1, #2, and #4), 2 genes chosen by GO terms (#21 and #27), and 1 gene chosen by degree (#19).

10.1128/msystems.00348-22.9 DATA SET S3 Nucleotide sequences of target genes. Download Data Set S3, XLSX file, 0.03 MB. Copyright © 2022 Walker et al. 2022 Walker et al. https://creativecommons.org/licenses/by/4.0/ This content is distributed under the terms of the Creative Commons Attribution 4.0 International license.

Six genes exhibited a statistical increase in cell mass in this high concentration of IL (#23, #10, #9, #25, #8, and #28). These gene targets were selected from only 2 target groups of overlap by degree and GO association (Fig. 3D). The 3 genes conferring enhanced IL tolerance chosen from overlap by degree included a membrane traffic protein (#8, TRS23, YALI0B22396g), a kinase activator (#9, CLB5, YALI0B15180g), and a microtubule binding motor protein (#10, KIP1/CIN8, YALI0F02673g). The 3 genes exhibiting increased IL robustness selected by GO terms were annotated as an integral component of the membrane (#23, YALI0B12738g), a protein kinase (#25, SPS1, YALI0D19470g), and a myosin heavy chain (#28, MYO1, YALI0F13343g).

### (ii) Dual-gene library enrichment.

**FIG 4:**
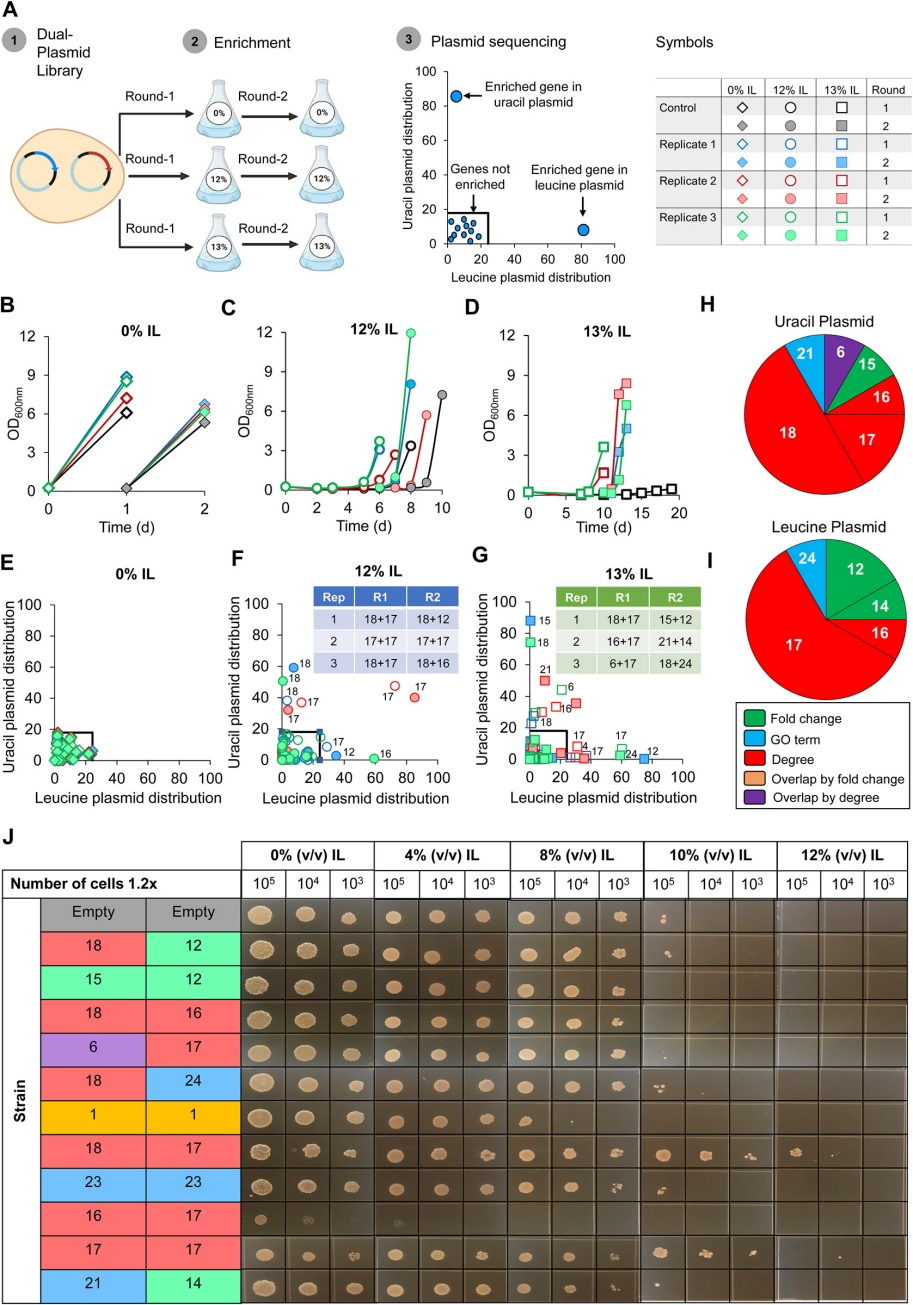
(A) Workflow of dual-gene library enrichment and plasmid sequencing (created with BioRender.com). (B to D) Growth of empty-vector control and library cocktail replicates in 0% (vol/vol) IL (B), 12% (vol/vol) IL (C), and 13% (vol/vol) IL (D). (E to G) Plasmid sequencing to identify enriched combinations of genes for library-cocktail replicates in 0% (vol/vol) IL (E), 12% (vol/vol) IL (F), and 13% (vol/vol) IL (G). (H and I) Frequency of enriched genes from library-cocktail enrichment selected by fold change (green), GO term (blue), degree (red), overlap by fold change (yellow), and overlap by degree (purple). (J) Plate spotting characterization of enriched combinations of genes from library-cocktail enrichment on 0%, 4%, 8%, 10%, and 12% (vol/vol) IL.

To identify the most IL-tolerant combination of genes, we duplicated the 29 gene target overexpression plasmids with a different selection marker (i.e., leucine and uracil selection markers) and combined the 2 sets of 29 overexpression plasmids into a library cocktail stock at equimolar concentrations (Fig. 4A). This library was transformed into Y. lipolytica, and 3 biological replicate transformants were cultured in 0%, 12%, and 13% (vol/vol) IL for 2 rounds. While all strains grew quickly in 0% IL, cell growth was delayed for several days in the high IL concentrations (Fig. 4B to D). Nevertheless, the dual-plasmid cocktail replicates grew faster than the empty-vector control in 12% IL (Fig. 4C), and all replicates were able to grow in 13% IL while the empty-vector control showed no growth in this high concentration of IL (Fig. 4D). Each cocktail replicate was sequenced after round 1 and round 2 cultures in 0%, 12%, and 13% IL to identify enriched combinations of genes selected from the challenging pressure of IL (Data Set S4).

10.1128/msystems.00348-22.10 DATA SET S4 Target dual-gene enrichment. Download Data Set S4, XLSX file, 0.02 MB. Copyright © 2022 Walker et al. 2022 Walker et al. https://creativecommons.org/licenses/by/4.0/ This content is distributed under the terms of the Creative Commons Attribution 4.0 International license.

For all replicates cultured in 0% IL, genes were not enriched beyond 24.3% of the total gene pool in the leucine plasmid and 18% of the total gene pool in the uracil plasmid (Fig. 4E). Plasmids sequenced from all replicate cultures in 12% and 13% IL revealed enriched combinations of genes (Fig. 4F and G). We found that replicate 2 cultured in 12% IL was the only replicate that maintained the same combination of genes between round 1 and round 2 (i.e., gene combination #17+#17) (Fig. 4F). However, gene combination #18+#17 was enriched in multiple replicates/rounds (i.e., 12% IL, replicate 1, round 1; 12% IL, replicate 3, round 1; and 13% IL, replicate 1, round 1). From these 9 combinations of genes, genes chosen by degree had the highest frequency in both the uracil plasmid (75% of genes) and leucine plasmid (66.7% of genes) (Fig. 4H and I). Notably, genes chosen by fold change showed the second highest frequency (8.3% of genes in uracil plasmid; 25% of genes in leucine plasmid) followed by genes chosen by GO term (8.3% of genes in both uracil and leucine plasmids) (Fig. 4H and I).

### (iii) Characterization of enriched gene combinations for IL tolerance.

**FIG 5:**
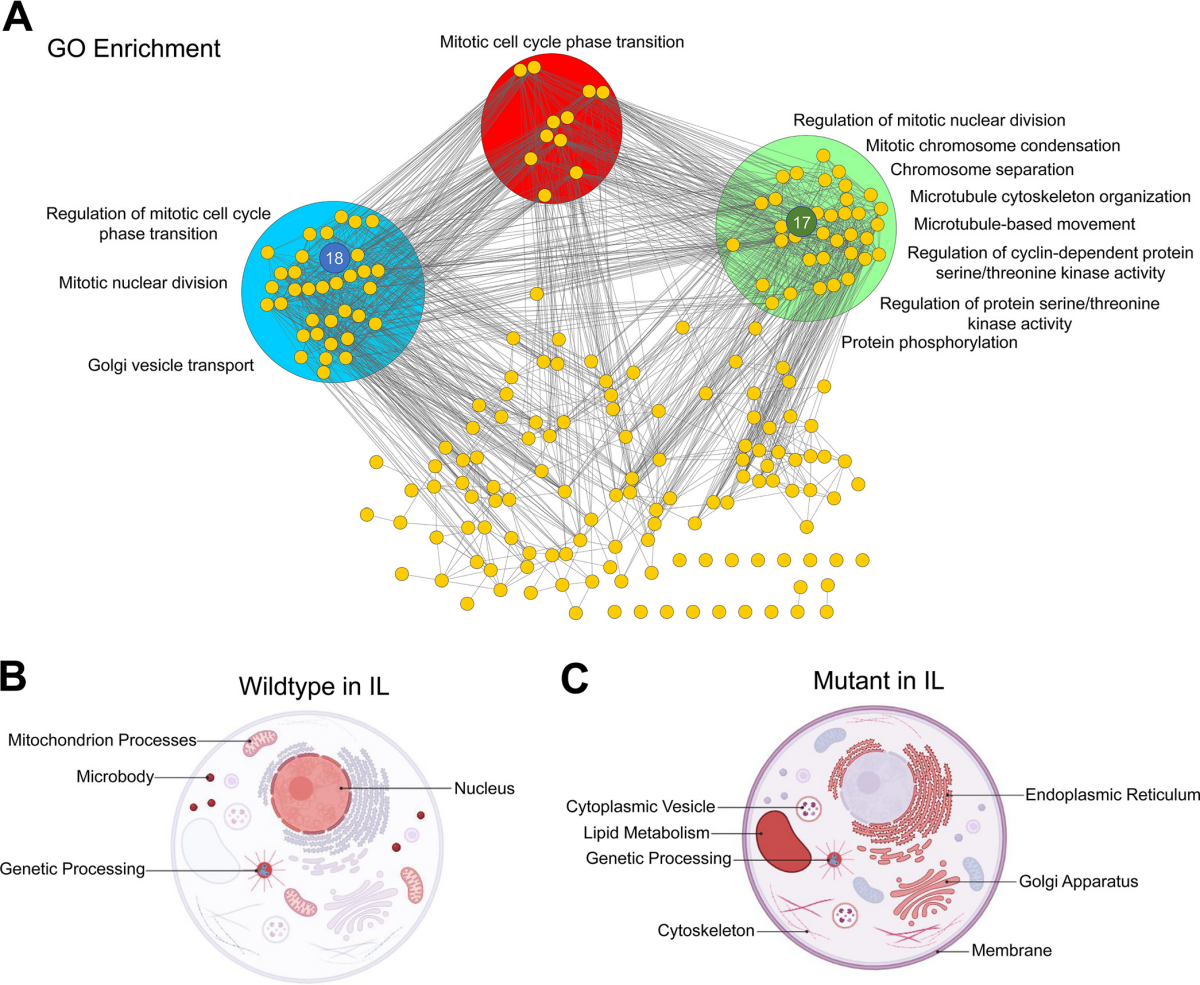
(A) Gene coexpression clusters for the top performing genes #17 and #18. (B and C) Cell model diagrams for top-performing genes when overexpressed individually (#8, #9, #10, #23, #25, and #28) and simultaneously (#17+#18) (A) for (A) WT and (B) MT in presence of IL. Panels B and C were created with BioRender.com.

Seeking to reverse engineer IL tolerance, we recreated strains individually bearing these 9 combinations of genes. For positive and negative controls, we generated strains bearing 2 copies of the best and worst genes from single-overexpression characterization (i.e., #23+#23 and #1+#1) along with the empty-vector control. Remarkably, gene combination #18+#17 (which was enriched in multiple replicates) demonstrated significantly higher IL tolerance relative to all strains tested (Fig. 4J). Interestingly, these genes were not directly coexpressed (i.e., not connected by an edge) but instead highly connected to other genes in the coexpression network. Gene ontology enrichment of the gene #17 cluster (genes connected to #17) and the gene #18 cluster (genes connected to #18), using the GO-slim biological process, revealed both gene clusters were enriched in mitotic nuclear division (Fig. 5A). The gene #17 cluster was also enriched for chromosome separation and mitotic chromosome condensation, microtubule cytoskeletal organization and microtubule-based movement, regulation of cyclin-dependent protein serine/threonine kinase activity, and protein phosphorylation (Fig. 5A). For the gene #18 cluster, the only uniquely enriched term was Golgi vesicle transport (Fig. 5A). The second-best strain contained the gene combination #17+#17, which was enriched in both rounds of 12% IL for replicate 2 (Fig. 4F and J). Gene combination #1+#1, which showed increased sensitivity to IL in single-overexpression characterization, was the worst-performing strain excluding gene combination #16+#17, which exhibited defective growth even when cultured without IL (Fig. 4J). Taken together, dual-plasmid library enrichment revealed genes #18 and #17, both chosen by degree (i.e., connectivity), which conferred high IL tolerance when overexpressed simultaneously.

## DISCUSSION

ILs inhibit microbial catalytic activity by affecting numerous cellular processes and components, which makes IL tolerance a difficult phenotype to engineer. Currently, reverse engineering complex phenotypes requires extensive characterization to understand key mechanisms and often leads to numerous gene targets for further experimentation (24–26). Here, we developed GeCCo to identify a subset of promising gene targets for reverse engineering IL tolerance by exploiting a superior IL-tolerant MT strain’s gene coexpression connectivity. From the pool of genes upregulated by the MT in IL (relative to the WT in IL at both exponential time points), we discovered that the best single-gene targets (i.e., single-gene overexpression) were predicted by gene connectivity overlapping in the 75th percentile of fold change (i.e., overlap by degree) and the best combination of gene targets (i.e., dual-gene overexpression) was predicted by coexpression connectivity alone (i.e., by degree).

Upon exposure to IL, our result shows that only the WT increased expression of genes associated with mitochondrion processes (i.e., respirasome and mitochondrial transmembrane transport) (Fig. 2J and Fig. 5B), suggesting ILs hinder mitochondrial function and oxidative phosphorylation. This result is consistent with a previously reported observation that ILs interfered with ion transport across the mitochondrial membrane, negatively affecting mitochondrial function and membrane potential in S. cerevisiae (27). In contrast, the WT repressed genes involved in cell wall organization/biogenesis (Fig. 2J), which vindicates the reduced cell wall chitin content when Y. lipolytica is exposed to IL (22).

The MT strain, however, induced expression of genes associated with lipid metabolism in IL (e.g., membrane, endoplasmic reticulum, Golgi apparatus, membrane traffic proteins, cytoplasmic vesicle, etc.) (Fig. 2K and Fig. 5C), which is supported by our previous discovery that biogenesis of sterols is critical for IL tolerance in Y. lipolytica (22). These results postulate other lipid metabolic processes (e.g., vesicle-mediated transport) underlying the superior IL tolerance of the MT strain as seen from the drastic remodeling of lipid composition in IL (22). Both WT and MT strains responded to IL by increasing expression of genes associated with genetic processing (i.e., chromatin binding/regulation, chromosome, transcription factors, and signal transduction) (Fig. 2J and K and Fig. 5B and C), indicating ILs severely influence gene regulation and signal transduction.

Aiming to understand the genotype(s) conferring high solvent tolerance, we reasoned that the best gene candidates would be genes upregulated by the MT relative to the WT when cultured in IL. Most of the gene targets, when individually overexpressed, increased cell mass in IL, indicating that our strategy of exploiting genes expressed more by the MT strain in this comparison was successful (Fig. 2L), though we did not test genes from other pairwise comparisons (e.g., WT in 8% IL versus WT in 0% IL). Strikingly, coexpression connectivity in conjunction with fold change (i.e., overlap by degree) had the highest statistical prediction of IL-tolerant genes when overexpressed individually (Fig. 3E).

Even though the 6 top-performing single-gene targets have not previously been characterized in Y. lipolytica, these genes have orthologs in the model yeast S. cerevisiae except for gene #23. Gene #8 (TRS23, YALI0B22396g) is associated with regulation of traffic from the endoplasmic reticulum to the Golgi apparatus and autophagy (28). Gene #9 (CLB5, YALI0B15180g) is a nonessential B-type cyclin involved in replication of DNA (29) whereby deletion increases sensitivity to rapamycin, caffeine, hydroxyurea, doxorubicin, and many other chemicals (30–32). Gene #10 (CIN8, YALI0F02673g) is a nonessential kinesin motor protein with involvement in chromosome segregation and in mitotic spindle assembly (33). Notably, CIN8-deleted S. cerevisiae exhibited abnormally large cell size, poor growth, increased mitophagy, and increased sensitivity to heat and various chemicals (34). Gene #25 (SPS1, YALI0D19470g) is a kinase regulator essential for prospore membrane closure (35). Gene #28 (MYO1, YALI0F13343g) is a class II myosin heavy chain with a key role in cytokinesis (36). Deletion of MYO1 in S. cerevisiae decreases resistance to nikkomycin Z, an antibiotic that inhibits chitin synthase (37).

The dual-gene overexpression library enrichment revealed combinations of genes that differed from the top-performing individual overexpression results (Fig. 4F and G). Most of the enriched gene targets in these dual-gene combinations were those selected by coexpression connectivity (i.e., degree) (Fig. 4H and I), indicating interdependency between highly connected genes. This notion is further supported by the significant increase in IL tolerance when #17 and #18 were simultaneously overexpressed (Fig. 4J) relative to the IL tolerance of either gene’s individual overexpression (Fig. 3D).

Notably, these genes are not obvious targets to reverse engineer IL tolerance based on annotations alone. Gene #17 (BRN1, YALI0B03476g) is an essential gene in S. cerevisiae required for the mitotic chromosome segregation and condensation (38). Gene #18 (OYE2, YALI0D16247g) is a flavin mononucleotide oxidoreductase that mediates resistance to small alpha- and beta-unsaturated carbonyls such as acrolein, a product of lipid peroxidation (39), and increases abundance in response to DNA replication stress (40). Further, OYE2 (gene #18) has been shown to be an antioxidant protein in S. cerevisiae, and overexpression of OYE2 (gene #18) rescues cell death induced by Bax, a proapoptotic protein that causes hyperpolarization of the mitochondrial membrane resulting in liberation of cytochrome *c* and reactive oxygen species (41). While lacking a strong connection as to how these two genes confer IL tolerance together, we can deduce that both genes are involved in the mutual process of mitotic cell cycle phase transition based on their coexpression gene clusters (Fig. 5A). Further, we can reason that IL toxicity is negatively affecting chromatin segregation during mitosis and that proteins responsible for guiding and regulating these processes (i.e., microtubule and cytoskeletal organization, kinases, and Golgi vesicle transport) are critical (Fig. 5A).

While coexpression networks have been used to infer gene function, gene association, and gene regulation, this is the first study to our knowledge where coexpression was used as a metric to select gene targets for reverse engineering. Though limited by the number of genes, phenotypes, and organisms tested in our study, these results highlight GeCCo as a promising selection tool to predict gene targets for reverse engineering complex phenotypes.

## MATERIALS AND METHODS

### Strains.

The Y. lipolytica strain, ATCC MYA-2613, a thiamine, leucine, and uracil auxotroph, was purchased from the American Type Culture Collection and used as the parent strain (WT). The evolved strain YlCW001 (MT) was isolated after 200 generations in gradually increased concentrations of [EMIM][OAc] up to 18% (vol/vol) (22). The TOP10 Escherichia coli strain was used for molecular cloning. IL-gene-overexpressing strains were generated by transforming constructed plasmids into WT via electroporation (42).

### Plasmids.

The plasmid pSR001 (21), harboring a leucine selection marker, TEF promoter, and CYC1 terminator, was used as the backbone plasmid for individual IL-gene overexpression cassettes. The plasmid pSR008 (43), identical to pSR001 except for the selection marker being replaced with uracil, was used for dual-gene overexpression. Both pSR001 and pSR008 were linearized by PCR amplification using primers 2 and 3, and each gene target was amplified from Y. lipolytica genomic DNA using the primers 79 to 136 listed in Table S2 in the supplemental material. Nucleotide sequences of the 29 target genes can be found in Data Set S3. Plasmids were constructed with Gibson assembly (44) using the linearized pSR001/pSR008 backbones and each amplified IL gene. The complete list of strains and plasmids is given in Table S3.

10.1128/msystems.00348-22.3 TABLE S2 Primers used for constructing gene overexpression plasmids. Download Table S2, XLSX file, 0.01 MB. Copyright © 2022 Walker et al. 2022 Walker et al. https://creativecommons.org/licenses/by/4.0/ This content is distributed under the terms of the Creative Commons Attribution 4.0 International license.

10.1128/msystems.00348-22.4 TABLE S3 Strains and plasmids used for single- and dual-gene overexpression. Download Table S3, XLSX file, 0.01 MB. Copyright © 2022 Walker et al. 2022 Walker et al. https://creativecommons.org/licenses/by/4.0/ This content is distributed under the terms of the Creative Commons Attribution 4.0 International license.

### Media and culturing conditions. (i) Growth medium.

Defined minimal medium contained 6.7 g/L of yeast nitrogen base without amino acids (catalog no. Y0626; Sigma-Aldrich, MO, USA), 20 g/L of glucose, 380 mg/L leucine (catalog no. 172130250; Acros Organics, CA, USA), 76 mg/L uracil (catalog no. 157301000; Acros Organics, CA, USA), and various concentrations of [EMIM][OAc] (>95% purity) (IoLiTec, AL, USA). Synthetic complete medium without leucine and uracil (SC-Leu-Ura) was prepared with 6.7 g/L of yeast nitrogen base without amino acids; 1.46 g/L of yeast synthetic dropout medium supplement without uracil, leucine, and tryptophan (catalog no. Y1771; Sigma-Aldrich, MO, USA); 76 mg/L tryptophan (catalog no. 172110250; Acros Organics, CA, USA), 20 g/L glucose, and various concentrations of [EMIM][OAc]. SC without leucine (SC-Leu) was prepared by adding 76 mg/L uracil to SC-Leu-Ura medium.

### (ii) Culturing conditions.

Seed cultures were conducted by inoculating a single colony from a fresh petri dish into 2 mL of medium using a 14-mL culture tube and incubated overnight in a MaxQ6000 air incubator set at 28°C and 250 rpm. Subcultures were conducted by transferring 1 mL of seed cultures into 10 mL of medium using 50-mL baffled flasks and cultured overnight until mid-exponential growth phase. The main experiment cultures were performed using 50-mL baffled flasks with 10 mL of medium in 3 technical replicates per biological condition and repeated to achieve a minimum of 6 replicates.

### (iii) Single-gene overexpression characterization.

Single-gene overexpression experiments were conducted using SC-Leu medium containing 11% (vol/vol) [EMIM][OAc] with an inoculum with a value of optical density at 600 nm (OD_600_) of 0.25 from subseed cultures.

### (iv) Dual-gene enrichment.

Equimolar concentrations of all 29 gene overexpression plasmids with a leucine selection marker and all 29 gene overexpression plasmids with a uracil selection marker were combined into a library cocktail. The library cocktail was transformed into WT Y. lipolytica, and recovered transformant colonies were combined and cultured in SC-Leu-Ura medium. Round 1 of the enrichment experiment was conducted in 50 mL of SC-Leu-Ura medium containing 0%, 12%, or 13% (vol/vol) [EMIM][OAc] in 3 replicates alongside a strain bearing an empty-vector leucine plasmid and an empty-vector uracil plasmid (i.e., control). Once cells achieved an OD_600_ above 3, the culture broth was centrifuged, resuspended in water, and used for inoculation in round 2 of enrichment before remaining cells were stored at −80°C. Once cells achieved an OD_600_ above 3, round 2 cultures were immediately stored at −80°C. Plasmids were extracted after thawing cells to room temperature using the Invitrogen ChargeSwitch plasmid yeast minikit (Fisher catalog no. CS10203).

### (v) Dual-gene plate screening.

Dual-gene overexpression characterization was performed with SC-Leu-Ura medium using subseed cultures that were centrifuged and resuspended to achieve an OD_600_ of 2 and diluted further by 10× and 100×. Two microliters of each dilution was transferred into gridded petri dishes containing SC-Leu-Ura medium, 20 g/L agar, and 0%, 4%, 8%, 10%, or 12% (vol/vol) [EMIM][OAc] and incubated at 28°C for 2, 3, 5, 10, and 10 days for each [EMIM][OAc] concentration, respectively. Plate screening was performed a minimum of 3 times for each construct.

### Analytical methods and bioinformatics. (i) Variant detection (mutation analysis).

Whole-genome resequencing reads of WT (GCA_009372015.1) and MT (GCA_009194645.1) strains were quality filtered as previously described (45) and imported to the CLC genomic workbench software version 11.0.1 (https://www.qiagenbioinformatics.com/). For each strain, the Map Reads to Reference tool was used with default parameters to map reads to reference genome Clib122 (GCA_000002525.1) (46). Variants for each strain were identified using the InDels and Structural Variants 1.81, Local Realignment 0.41, and Fixed Ploidy Variant Detection 1.67 tools all set with default parameters. Variants specific to the MT strain were identified using the Compare Sample Variant Tracks 1.4 tool, and variants causing an amino acid change were identified using the Amino Acid Changes 2.4 tool. Types of nucleotide changes were classified in different categories including single nucleotide variants (SNV), multiple nucleotide variants (MNV), insertions, deletions, and replacements.

### (ii) RNA sequencing.

Samples were collected in biological triplicates for RNA sequencing from WT and MT strains at early and mid-exponential growth phases in 0% and 8% (vol/vol) [EMIM][OAc]. Samples were immediately quenched in liquid nitrogen and stored at −80°C until RNA isolation. Total RNA was purified using the Qiagen RNeasy minikit (catalog no. 74104; Qiagen, CA, USA) prior to submission at the Joint Genome Institute (JGI) using Illumina sequencing. Filtered RNA-Seq reads were analyzed within the CLC genomics workbench version 11.0.1 (https://www.qiagenbioinformatics.com/) using the RNA-Seq analysis tool to produce transcripts-per-million (TPM) values for each gene/sample that were used for all downstream transcript analyses (see Table S4 in the supplemental material).

10.1128/msystems.00348-22.5 TABLE S4 Transcript-per-million (TPM) values for both WT and MT Y. lipolytica strains growing in 0% and 8% IL. Download Table S4, XLSX file, 0.9 MB. Copyright © 2022 Walker et al. 2022 Walker et al. https://creativecommons.org/licenses/by/4.0/ This content is distributed under the terms of the Creative Commons Attribution 4.0 International license.

### (iii) Plasmid amplification and Oxford Nanopore sequencing.

Extracted plasmids were amplified using random hexamer primers (Thermo Scientific SO181) and ϕ29 DNA polymerase (NEB M0269L), as previously described (47), with the exception of using the commercial ϕ29 reaction buffer from NEB (B0269L). Amplification products were cleaned and concentrated using the E.Z.N.A. Cycle-Pure kit from Omega BioTek (D6492-02) and quantified using Nanodrop.

Purified DNA was then sequenced using Oxford Nanopore’s ligation sequencing chemistry (SQK-LSK109) in conjunction with native barcoding expansions (EXP-NBD104/114) for high throughput, according to the manufacturer’s recommendations. All samples were sequenced on a MinION device using R9.4.1 flow cells. Samples were demultiplexed and base called either (i) in real time with MinKNOW v4.3.20 using the fast base-calling algorithm or (ii) asynchronously with Guppy GPU v5.0.11 on Ubuntu 20.04 LTS using the fast base-calling algorithm.

### (iv) Plasmid sequence mapping.

To call reads for certain gene/selection marker combinations in a sample, the demultiplexed FASTQ files were concatenated into a single file and run through Centrifuge (v 1.0.3) (48) in two iterations: (i) using the uracil and leucine selection marker sequences as the “genomes” for alignment and then (ii) using the 29 gene sequences as a basis for alignment (Data Set S3). Centrifuge then output .tsv files with each unique read ID that matched the sequence provided, using the standard algorithm alignment cutoff parameters. The two output files were cross-referenced for reads that hit on both the selection marker and a gene. The number of reads for each combination of gene/selection marker was tabulated for each sample (Data Set S4). The number of reads for each gene was divided by the total number of reads (which mapped to genes and respective selection marker) for each sample’s uracil plasmid and leucine plasmid separately. This value was multiplied by 100 to represent the percentage of individual gene reads of the total reads for each plasmid in each sample.

### (v) Gene ontology enrichment.

Gene ontology (GO) for genes containing variants identified from resequencing (i.e., mutated genes) was identified using ClueGo (49), Integrated Microbial Genomes (IMG) (50), and BLASTp against the model yeast S. cerevisiae with an E value of <1E−10. Annotations were combined and curated manually to classify mutated gene ontology. GO terms associated with classified genes were manually retrieved from the Panther database (51) (Table S5) and mapped to classified genes to calculate the number of genes associated with each annotation term. GO terms were defined as enriched if the sum of upregulated and increasing genes in an annotation term was equal to or greater than double the sum of upregulated and increasing genes between the two biological conditions in each pairwise data set. GO enrichment for gene clusters connected to gene #17 and gene #18 was conducted using the Panther overrepresentation test (http://geneontology.org/) (52–54) with the GO-slim biological process database and default settings (Fisher’s exact, calculate false-discovery rate), and parent terms were used for illustration.

10.1128/msystems.00348-22.6 TABLE S5 Panther GO terms for Y. lipolytica. Download Table S5, XLSX file, 0.3 MB. Copyright © 2022 Walker et al. 2022 Walker et al. https://creativecommons.org/licenses/by/4.0/ This content is distributed under the terms of the Creative Commons Attribution 4.0 International license.

### (vi) Statistics.

One-way analysis of variance (ANOVA) with Holm-Sidak correction was used for all statistical analyses with the SigmaPlot v.14 software.

### Gene coexpression connectivity (GeCCo) analysis. (i) Gene expression classification.

Gene TPM values were floored to a value of 5 and averaged into log_2_-scaled values to compare biological conditions (BC) in the following pairwise sets: (i) MT in 0% IL versus WT in 0% IL (BC1), (ii) WT in 8% IL versus WT in 0% IL (BC2), (iii) MT in 8% IL versus MT in 0% IL (BC3), and (iv) MT in 8% IL versus WT in 8% IL (BC4) (see Fig. S1, step 1). For each pairwise set, *X* score (i.e., early exponential fold change, equation 1), *Y* score (i.e., mid-exponential fold change, equation 2), and *Z* score (i.e., genes that are regulated differently during exponential growth, equation 3) were calculated for each gene as shown below (also see Fig. S1, step 2):

(1) $$X=\frac{\text{BC}1_{\text{early}}-\text{BC}2_{\text{early}}}{\sqrt{0.25+{\left(\frac{V}{N}\right)}_{\text{BC}1_{\text{early}}}+{\left(\frac{V}{N}\right)}_{{\text{BC2}}_{\text{early}}}}}$$

(2) $$Y=\frac{{\text{BC1}}_{\text{mid}}-{\text{BC2}}_{\text{mid}}}{\sqrt{0.25+{\left(\frac{V}{N}\right)}_{{\text{BC1}}_{\text{mid}}}+{\left(\frac{V}{N}\right)}_{{\text{BC2}}_{\text{mid}}}}}$$

(3) $$Z=\frac{\left({\text{BC1}}_{\text{mid}}-{\text{BC1}}_{\text{early}}\right)-({\text{BC2}}_{\text{mid}}-{\text{BC2}}_{\text{early}})}{\sqrt{0.25+{\left(\frac{V}{N}\right)}_{{\text{BC1}}_{\text{mid}}}+{\left(\frac{V}{N}\right)}_{{\text{BC1}}_{\text{early}}}+{\left(\frac{V}{N}\right)}_{{\text{BC2}}_{\text{mid}}}+{\left(N\right)}_{{\text{BC2}}_{\text{early}}}}}$$

where BC1 and BC2 are average log_2_(TPM) values of a gene, subscripts “early” and “mid” refer to early- and mid-exponential samples, respectively, *V* is variance, and *N* (=3) is the number of replicates. For each equation, the numerator calculates fold changes while the denominator accounts for error by dividing the fold change by the square root of pseudovariance (0.25) and the summation of variance (*V*) divided by respective number of replicates for each sample in the numerator.

Next, genes were categorized into 1 of 6 classes based on the values of *X*, *Y*, and *Z* scores, including BC1 upregulated (*X* ≥ 1 and *Y* ≥ 1), BC1 increasing (−1 < *X* < 1, *Y* ≥ 1, and *Z* ≥ 1.5), BC2 upregulated (*X* ≤ −1, *Y* ≤ −1), BC2 increasing (−1 < *X* < 1, *Y* ≤ −1, and *Z* ≤ −1.5), changed regulation (|*Z*| ≥ 1.5), and no change (Fig. S1, step 3). The “upregulated” gene class represents genes with greater expression values at both early- and mid-exponential samples. The “increasing” gene class represents genes without a significant change in expression value at the early-exponential sample but a greater expression value at mid-exponential sample. The “changed regulation” gene class represents genes that are neither upregulated nor increasing but with a regulation score greater than 1.5. The “no change” gene class represents genes that fail to meet the requirements of upregulated, increasing, or changed regulation classifications.

### (ii) Gene coexpression network construction and gene target selection.

Pearson correlation was conducted on early- and mid-exponential WT 8% IL and MT 8% IL sample-gene TPM values using a stringent cutoff of 0.95. Next, this coexpression network was reduced by removing all genes except those classified as upregulated by the MT 8% IL in pairwise set iv (i.e., MT 8% IL versus WT 8% IL) and used to calculate degree centrality. These genes were ranked according to their degree and their average fold change between the two transcriptomic time points (i.e., average of *X* and *Y* scores) and the 75th percentiles were calculated for both criteria. Finally, we chose 29 gene targets from the top 5 genes ranked by fold change, the top 5 genes by degree, the top 5 genes by degree overlapping fold change, the top 5 genes by fold change overlapping degree, and 9 genes with relevant gene ontology terms (e.g., membrane, kinase, transport, cell wall, and myosin complex).

GeCCo can be found at https://github.com/TrinhLab/GeCCo/.

## References

[1] Kim P-Y, Pollard DJ, Woodley JM. 2007. Substrate supply for effective biocatalysis. Biotechnol Prog 23:74–82. doi:10.1021/bp060314b. 17269674

[2] Klibanov AM. 2001. Improving enzymes by using them in organic solvents. Nature 409:241–246. doi:10.1038/35051719. 11196652

[3] Bruce LJ, Daugulis AJ. 1991. Solvent selection strategies for extractive biocatalysis. Biotechnol Prog 7:116–124. doi:10.1021/bp00008a006. 1367167

[4] Itoh T. 2017. Biotransformation in ionic liquid, p 27–67. *In* Matsuda T (ed), Future directions in biocatalysis, 2nd ed. Elsevier, Amsterdam, Netherlands.

[5] Moniruzzaman M, Nakashima K, Kamiya N, Goto M. 2010. Recent advances of enzymatic reactions in ionic liquids. Biochem Eng J 48:295–314. doi:10.1016/j.bej.2009.10.002.

[6] Li C, Knierim B, Manisseri C, Arora R, Scheller HV, Auer M, Vogel KP, Simmons BA, Singh S. 2010. Comparison of dilute acid and ionic liquid pretreatment of switchgrass: biomass recalcitrance, delignification and enzymatic saccharification. Bioresour Technol 101:4900–4906. doi:10.1016/j.biortech.2009.10.066. 19945861

[7] Yoo CG, Pu Y, Ragauskas AJ. 2017. Ionic liquids: promising green solvents for lignocellulosic biomass utilization. Curr Opin Green Sustain Chem 5:5–11. doi:10.1016/j.cogsc.2017.03.003.

[8] Socha AM, Parthasarathi R, Shi J, Pattathil S, Whyte D, Bergeron M, George A, Tran K, Stavila V, Venkatachalam S, Hahn MG, Simmons BA, Singh S. 2014. Efficient biomass pretreatment using ionic liquids derived from lignin and hemicellulose. Proc Natl Acad Sci USA 111:E3587–E3595. doi:10.1073/pnas.1405685111. 25136131 PMC4156760

[9] Mohamed ET, Wang S, Lennen RM, Herrgård MJ, Simmons BA, Singer SW, Feist AM. 2017. Generation of a platform strain for ionic liquid tolerance using adaptive laboratory evolution. Microb Cell Fact 16:204. doi:10.1186/s12934-017-0819-1. 29145855 PMC5691611

[10] Frederix M, Mingardon F, Hu M, Sun N, Pray T, Singh S, Simmons BA, Keasling JD, Mukhopadhyay A. 2016. Development of an E. coli strain for one-pot biofuel production from ionic liquid pretreated cellulose and switchgrass. Green Chem 18:4189–4197. doi:10.1039/C6GC00642F.

[11] Wu Y-W, Higgins B, Yu C, Reddy AP, Ceballos S, Joh LD, Simmons BA, Singer SW, VanderGheynst JS. 2016. Ionic liquids impact the bioenergy feedstock-degrading microbiome and transcription of enzymes relevant to polysaccharide hydrolysis. mSystems 1:e00120-16. doi:10.1128/mSystems.00120-16. 27981239 PMC5155067

[12] Ouellet M, Datta S, Dibble DC, Tamrakar PR, Benke PI, Li C, Singh S, Sale KL, Adams PD, Keasling JD, Simmons BA, Holmes BM, Mukhopadhyay A. 2011. Impact of ionic liquid pretreated plant biomass on *Saccharomyces cerevisiae* growth and biofuel production. Green Chem 13:2743–2749. doi:10.1039/c1gc15327g.

[13] Yu M, Wang S-H, Luo Y-R, Han Y-W, Li X-Y, Zhang B-J, Wang J-J. 2009. Effects of the 1-alkyl-3-methylimidazolium bromide ionic liquids on the antioxidant defense system of *Daphnia magna*. Ecotoxicol Environ Saf 72:1798–1804. doi:10.1016/j.ecoenv.2009.05.002. 19501400

[14] Docherty KM, Kulpa JCF. 2005. Toxicity and antimicrobial activity of imidazolium and pyridinium ionic liquids. Green Chem 7:185–189. doi:10.1039/b419172b.

[15] Liu L-P, Zong M-H, Linhardt RJ, Lou W-Y, Li N, Huang C, Wu H. 2016. Mechanistic insights into the effect of imidazolium ionic liquid on lipid production by *Geotrichum fermentans*. Biotechnol Biofuels 9:266. doi:10.1186/s13068-016-0682-z. 28018484 PMC5162095

[16] Lim HG, Fong B, Alarcon G, Magurudeniya HD, Eng T, Szubin R, Olson CA, Palsson BO, Gladden JM, Simmons BA, Mukhopadhyay A, Singer SW, Feist AM. 2020. Generation of ionic liquid tolerant Pseudomonas putida KT2440 strains via adaptive laboratory evolution. Green Chem 22:5677–5690. doi:10.1039/D0GC01663B.

[17] Ruegg TL, Kim E-M, Simmons BA, Keasling JD, Singer SW, Lee TS, Thelen MP. 2014. An auto-inducible mechanism for ionic liquid resistance in microbial biofuel production. Nat Commun 5:3490. doi:10.1038/ncomms4490. 24667370

[18] Higgins DA, Young MKM, Tremaine M, Sardi M, Fletcher JM, Agnew M, Liu L, Dickinson Q, Peris D, Wrobel RL, Hittinger CT, Gasch AP, Singer SW, Simmons BA, Landick R, Thelen MP, Sato TK. 2018. Natural variation in the multidrug efflux pump SGE1 underlies ionic liquid tolerance in yeast. Genetics 210:219–234. doi:10.1534/genetics.118.301161. 30045857 PMC6116967

[19] Reed KB, Wagner JM, d’Oelsnitz S, Wiggers JM, Alper HS. 2019. Improving ionic liquid tolerance in Saccharomyces cerevisiae through heterologous expression and directed evolution of an ILT1 homolog from Yarrowia lipolytica. J Ind Microbiol Biotechnol 46:1715–1724. doi:10.1007/s10295-019-02228-9. 31428944

[20] Sitepu IR, Shi S, Simmons BA, Singer SW, Boundy-Mills K, Simmons CW. 2014. Yeast tolerance to the ionic liquid 1-ethyl-3-methylimidazolium acetate. FEMS Yeast Res 14:1286–1294. doi:10.1111/1567-1364.12224. 25348480

[21] Ryu S, Labbé N, Trinh CT. 2015. Simultaneous saccharification and fermentation of cellulose in ionic liquid for efficient production of α-ketoglutaric acid by Yarrowia lipolytica. Appl Microbiol Biotechnol 99:4237–4244. doi:10.1007/s00253-015-6521-5. 25783627

[22] Walker C, Ryu S, Trinh CT. 2019. Exceptional solvent tolerance in Yarrowia lipolytica is enhanced by sterols. Metab Eng 54:83–95. doi:10.1016/j.ymben.2019.03.003. 30885767

[23] Randhawa V, Pathania S. 2020. Advancing from protein interactomes and gene co-expression networks towards multi-omics-based composite networks: approaches for predicting and extracting biological knowledge. Brief Funct Genomics 19:364–376. doi:10.1093/bfgp/elaa015. 32678894

[24] Chen Y, Boggess EE, Ocasio ER, Warner A, Kerns L, Drapal V, Gossling C, Ross W, Gourse RL, Shao Z, Dickerson J, Mansell TJ, Jarboe LR. 2020. Reverse engineering of fatty acid-tolerant Escherichia coli identifies design strategies for robust microbial cell factories. Metab Eng 61:120–130. doi:10.1016/j.ymben.2020.05.001. 32474056 PMC7501233

[25] Mans R, Daran J-MG, Pronk JT. 2018. Under pressure: evolutionary engineering of yeast strains for improved performance in fuels and chemicals production. Curr Opin Biotechnol 50:47–56. doi:10.1016/j.copbio.2017.10.011. 29156423

[26] Choi KR, Jang WD, Yang D, Cho JS, Park D, Lee SY. 2019. Systems metabolic engineering strategies: integrating systems and synthetic biology with metabolic engineering. Trends Biotechnol 37:817–837. doi:10.1016/j.tibtech.2019.01.003. 30737009

[27] Dickinson Q, Bottoms S, Hinchman L, McIlwain S, Li S, Myers CL, Boone C, Coon JJ, Hebert A, Sato TK, Landick R, Piotrowski JS. 2016. Mechanism of imidazolium ionic liquids toxicity in Saccharomyces cerevisiae and rational engineering of a tolerant, xylose-fermenting strain. Microb Cell Fact 15:17. doi:10.1186/s12934-016-0417-7. 26790958 PMC4721058

[28] Sacher M, Barrowman J, Schieltz D, Yates JR, Ferro-Novick S. 2000. Identification and characterization of five new subunits of TRAPP. Eur J Cell Biol 79:71–80. doi:10.1078/S0171-9335(04)70009-6. 10727015

[29] Schwob E, Nasmyth K. 1993. CLB5 and CLB6, a new pair of B cyclins involved in DNA replication in Saccharomyces cerevisiae. Genes Dev 7:1160–1175. doi:10.1101/gad.7.7a.1160. 8319908

[30] Dudley AM, Janse DM, Tanay A, Shamir R, Church GM. 2005. A global view of pleiotropy and phenotypically derived gene function in yeast. Mol Syst Biol 1:2005.0001. doi:10.1038/msb4100004. PMC1681449 16729036

[31] Westmoreland TJ, Wickramasekara SM, Guo AY, Selim AL, Winsor TS, Greenleaf AL, Blackwell KL, Olson JA, Marks JR, Bennett CB. 2009. Comparative genome-wide screening identifies a conserved doxorubicin repair network that is diploid specific in Saccharomyces cerevisiae. PLoS One 4:e5830. doi:10.1371/journal.pone.0005830. 19503795 PMC2688081

[32] Kapitzky L, Beltrao P, Berens TJ, Gassner N, Zhou C, Wüster A, Wu J, Babu MM, Elledge SJ, Toczyski D, Lokey RS, Krogan NJ. 2010. Cross-species chemogenomic profiling reveals evolutionarily conserved drug mode of action. Mol Syst Biol 6:451. doi:10.1038/msb.2010.107. 21179023 PMC3018166

[33] Hoyt MA, He L, Loo KK, Saunders WS. 1992. Two Saccharomyces cerevisiae kinesin-related gene products required for mitotic spindle assembly. J Cell Biol 118:109–120. doi:10.1083/jcb.118.1.109. 1618897 PMC2289527

[34] Basmaji F, Martin-Yken H, Durand F, Dagkessamanskaia A, Pichereaux C, Rossignol M, Francois J. 2006. The ‘interactome’ of the Knr4/Smi1, a protein implicated in coordinating cell wall synthesis with bud emergence in Saccharomyces cerevisiae. Mol Genet Genomics 275:217–230. doi:10.1007/s00438-005-0082-8. 16362369

[35] Iwamoto MA, Fairclough SR, Rudge SA, Engebrecht J. 2005. Saccharomyces cerevisiae Sps1p regulates trafficking of enzymes required for spore wall synthesis. Eukaryot Cell 4:536–544. doi:10.1128/EC.4.3.536-544.2005. 15755916 PMC1087804

[36] Watts FZ, Shiels G, Orr E. 1987. The yeast MYO1 gene encoding a myosin-like protein required for cell division. EMBO J 6:3499–3505. doi:10.1002/j.1460-2075.1987.tb02675.x. 3322809 PMC553809

[37] Rivera-Molina FE, González-Crespo S, Maldonado-De la Cruz Y, Ortiz-Betancourt JM, Rodríguez-Medina JR. 2006. 2,3-Butanedione monoxime increases sensitivity to nikkomycin Z in the budding yeast Saccharomyces cerevisiae. World J Microbiol Biotechnol 22:255–260. doi:10.1007/s11274-005-9028-x. 25382940 PMC4222539

[38] Lavoie BD, Tuffo KM, Oh S, Koshland D, Holm C. 2000. Mitotic chromosome condensation requires Brn1p, the yeast homologue of Barren. Mol Biol Cell 11:1293–1304. doi:10.1091/mbc.11.4.1293. 10749930 PMC14847

[39] Trotter EW, Collinson EJ, Dawes IW, Grant CM. 2006. Old yellow enzymes protect against acrolein toxicity in the yeast *Saccharomyces cerevisiae*. Appl Environ Microbiol 72:4885–4892. doi:10.1128/AEM.00526-06. 16820484 PMC1489299

[40] Tkach JM, Yimit A, Lee AY, Riffle M, Costanzo M, Jaschob D, Hendry JA, Ou J, Moffat J, Boone C, Davis TN, Nislow C, Brown GW. 2012. Dissecting DNA damage response pathways by analysing protein localization and abundance changes during DNA replication stress. Nat Cell Biol 14:966–976. doi:10.1038/ncb2549. 22842922 PMC3434236

[41] Odat O, Matta S, Khalil H, Kampranis SC, Pfau R, Tsichlis PN, Makris AM. 2007. Old yellow enzymes, highly homologous FMN oxidoreductases with modulating roles in oxidative stress and programmed cell death in yeast. J Biol Chem 282:36010–36023. doi:10.1074/jbc.M704058200. 17897954

[42] Markham KA, Vazquez S, Alper HS. 2018. High-efficiency transformation of Yarrowia lipolytica using electroporation. FEMS Yeast Res 18 (7). doi:10.1093/femsyr/foy081. 30052958

[43] Ryu S, Trinh CT. 2018. Understanding functional roles of native pentose-specific transporters for activating dormant pentose metabolism in *Yarrowia lipolytica*. Appl Environ Microbiol 84:e02146-17. doi:10.1128/AEM.02146-17. 29150499 PMC5772232

[44] Gibson DG, Young L, Chuang R-Y, Venter JC, Hutchison CA, Smith HO. 2009. Enzymatic assembly of DNA molecules up to several hundred kilobases. Nat Methods 6:343–345. doi:10.1038/nmeth.1318. 19363495

[45] Walker C, Ryu S, Haridas S, Na H, Zane M, LaButti K, Barry K, Grigoriev IV, Trinh CT. 2020. Draft genome assemblies of ionic liquid-resistant *Yarrowia lipolytica* PO1f and its superior evolved strain, YlCW001. Microbiol Resour Announc 9:e01356-19. doi:10.1128/MRA.01356-19. 32107299 PMC7046820

[46] Dujon B, Sherman D, Fischer G, Durrens P, Casaregola S, Lafontaine I, De Montigny J, Marck C, Neuvéglise C, Talla E, Goffard N, Frangeul L, Aigle M, Anthouard V, Babour A, Barbe V, Barnay S, Blanchin S, Beckerich J-M, Beyne E, Bleykasten C, Boisramé A, Boyer J, Cattolico L, Confanioleri F, De Daruvar A, Despons L, Fabre E, Fairhead C, Ferry-Dumazet H, Groppi A, Hantraye F, Hennequin C, Jauniaux N, Joyet P, Kachouri R, Kerrest A, Koszul R, Lemaire M, Lesur I, Ma L, Muller H, Nicaud J-M, Nikolski M, Oztas S, Ozier-Kalogeropoulos O, Pellenz S, Potier S, Richard G-F, Straub M-L, Suleau A, Swennen D, Tekaia F, Wésolowski-Louvel M, Westhof E, Wirth B, Zeniou-Meyer M, Zivanovic I, Bolotin-Fukuhara M, Thierry A, Bouchier C, Caudron B, Scarpelli C, Gaillardin C, Weissenbach J, Wincker P, Souciet JL. 2004. Genome evolution in yeasts. Nature 430:35–44. doi:10.1038/nature02579. 15229592

[47] Dean FB, Hosono S, Fang L, Wu X, Faruqi AF, Bray-Ward P, Sun Z, Zong Q, Du Y, Du J, Driscoll M, Song W, Kingsmore SF, Egholm M, Lasken RS. 2002. Comprehensive human genome amplification using multiple displacement amplification. Proc Natl Acad Sci USA 99:5261–5266. doi:10.1073/pnas.082089499. 11959976 PMC122757

[48] Kim D, Song L, Breitwieser FP, Salzberg SL. 2016. Centrifuge: rapid and sensitive classification of metagenomic sequences. Genome Res 26:1721–1729. doi:10.1101/gr.210641.116. 27852649 PMC5131823

[49] Bindea G, Mlecnik B, Hackl H, Charoentong P, Tosolini M, Kirilovsky A, Fridman W-H, Pagès F, Trajanoski Z, Galon J. 2009. ClueGO: a Cytoscape plug-in to decipher functionally grouped gene ontology and pathway annotation networks. Bioinformatics 25:1091–1093. doi:10.1093/bioinformatics/btp101. 19237447 PMC2666812

[50] Chen I-MA, Chu K, Palaniappan K, Ratner A, Huang J, Huntemann M, Hajek P, Ritter S, Varghese N, Seshadri R, Roux S, Woyke T, Eloe-Fadrosh EA, Ivanova NN, Kyrpides NC. 2021. The IMG/M data management and analysis system v.6.0: new tools and advanced capabilities. Nucleic Acids Res 49:D751–D763. doi:10.1093/nar/gkaa939. 33119741 PMC7778900

[51] Mi H, Ebert D, Muruganujan A, Mills C, Albou L-P, Mushayamaha T, Thomas PD. 2021. PANTHER version 16: a revised family classification, tree-based classification tool, enhancer regions and extensive API. Nucleic Acids Res 49:D394–D403. doi:10.1093/nar/gkaa1106. 33290554 PMC7778891

[52] Ashburner M, Ball CA, Blake JA, Botstein D, Butler H, Cherry JM, Davis AP, Dolinski K, Dwight SS, Eppig JT, Harris MA, Hill DP, Issel-Tarver L, Kasarskis A, Lewis S, Matese JC, Richardson JE, Ringwald M, Rubin GM, Sherlock G. 2000. Gene Ontology: tool for the unification of biology. Nat Genet 25:25–29. doi:10.1038/75556. 10802651 PMC3037419

[53] Gene Ontology Consortium. 2021. The Gene Ontology resource: enriching a GOld mine. Nucleic Acids Res 49:D325–D334. doi:10.1093/nar/gkaa1113. 33290552 PMC7779012

[54] Mi H, Muruganujan A, Ebert D, Huang X, Thomas PD. 2019. PANTHER version 14: more genomes, a new PANTHER GO-slim and improvements in enrichment analysis tools. Nucleic Acids Res 47:D419–D426. doi:10.1093/nar/gky1038. 30407594 PMC6323939

