# Supplementary figures and images for: Gene Coexpression Connectivity Predicts Gene Targets Underlying High Ionic-Liquid Tolerance in Yarrowia lipolytica
Source: mSystems. 2022 Jul 12;7(4):e00348-22. doi: 10.1128/msystems.00348-22 (PMC9426553; doi:10.1128/msystems.00348-22)

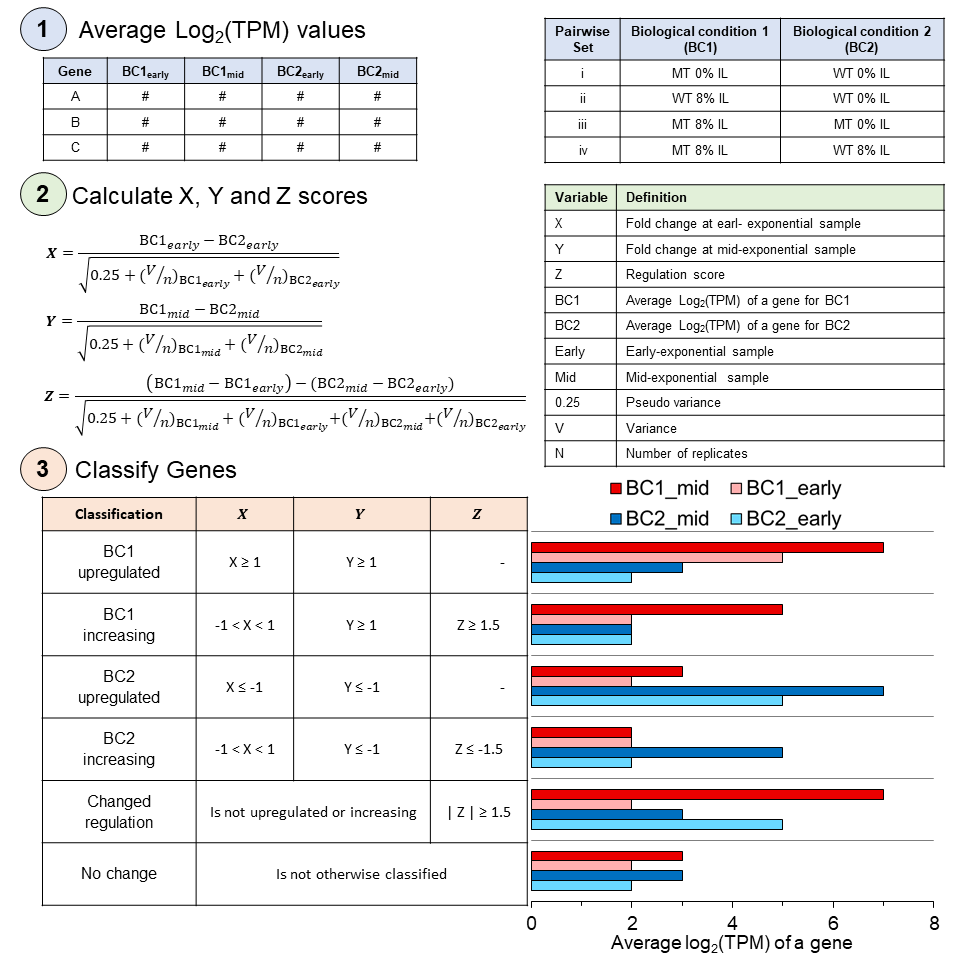

Supplement: FIG S1 [file msystems.00348-22-s0006.tif]
